# Validation of Inverse Seasonal Peak Mortality in Medieval Plagues, Including the Black Death, in Comparison to Modern *Yersinia pestis*-Variant Diseases

**DOI:** 10.1371/journal.pone.0008401

**Published:** 2009-12-22

**Authors:** Mark R. Welford, Brian H. Bossak

**Affiliations:** 1 Department of Geology and Geography, Georgia Southern University, Statesboro, Georgia, United States of America; 2 Jiann-Ping Hsu College of Public Health, Georgia Southern University, Statesboro, Georgia, United States of America; University of Sydney, Australia

## Abstract

**Background:**

Recent studies have noted myriad qualitative and quantitative inconsistencies between the medieval Black Death (and subsequent “plagues”) and modern empirical *Y. pestis* plague data, most of which is derived from the Indian and Chinese plague outbreaks of A.D. 1900±15 years. Previous works have noted apparent differences in seasonal mortality peaks during Black Death outbreaks versus peaks of bubonic and pneumonic plagues attributed to *Y. pestis* infection, but have not provided spatiotemporal statistical support. Our objective here was to validate individual observations of this seasonal discrepancy in peak mortality between historical epidemics and modern empirical data.

**Methodology/Principal Findings:**

We compiled and aggregated multiple daily, weekly and monthly datasets of both *Y. pestis* plague epidemics and suspected Black Death epidemics to compare seasonal differences in mortality peaks at a monthly resolution. Statistical and time series analyses of the epidemic data indicate that a seasonal inversion in peak mortality does exist between known *Y. pestis* plague and suspected Black Death epidemics. We provide possible explanations for this seasonal inversion.

**Conclusions/Significance:**

These results add further evidence of inconsistency between historical plagues, including the Black Death, and our current understanding of *Y. pestis*-variant disease. We expect that the line of inquiry into the disputed cause of the greatest recorded epidemic will continue to intensify. Given the rapid pace of environmental change in the modern world, it is crucial that we understand past lethal outbreaks as fully as possible in order to prepare for future deadly pandemics.

## Introduction

Recent reanalysis of medieval Black Death data (qualitative and quantitative) has resulted in a growing number of published works noting inconstancies between Y*ersinia pestis*-variant disease (bubonic and pneumonic plague) and its attribution to this historical pestilence [1], [2], [3]–[10]. Discrepancies have been noted pertaining to unsupported vector-host dynamics [1], [4], [5], [8], epidemic velocity and peak seasonality of mortality [2], [3], [5], [9], symptomatic descriptions [1], [5], [9], [10], spatiotemporal dynamics [2], [3], [7], and empirical observations of modern Y plagues [11]–[15] among other factors. Nevertheless, the paradigm of medieval Black Death causation (bubonic/pneumonic plague) adopted around the turn of the 20^th^ century remains the dominant etiologic theory.

One of the difficulties in supporting an alternative etiologic hypothesis for the Black Death is the uncertainty inherent in historical epidemic records and documentation. This uncertainty is unfortunate in that the temporal dynamics of an individual disease's epidemiological distribution can suggest transmission routes, seasonal peaks in virulence, and in replicate, a similar etiology. Yet detailed and clearly articulated descriptions of Black Death symptoms (that fail to match either bubonic or pneumonic plague) by doctors (and writers) of the period are known (e.g., Boccaccio in 1353 [16]; William Boghurst writing in 1666 [17]; Dr George Thompson describing an autopsy in 1666 [17]; Dr Nathaniel Hodges writing in 1666 [18]). In fact, by 1632, doctors in England had been asked to identify five different human-to-human infectious diseases (TB, Small Pox, Measles, French Pox, and Plague) when submitting information for the ‘Bills of Mortality’ [19].

In addition to the difficulties in reconstructing epidemiologic parameters from scattered historical quantitative data, suggesting disease etiology from historical non-empirical data (e.g., symptomatic descriptions) is even more difficult and suspect. Yet this is exactly what occurred in the late 19^th^ century based on symptomatic comparisons between an Asian epidemic of bubonic plague [20] and selected historical accounts of the medieval Black Death. Nonetheless, historical records can be of use in extracting defensible information pertaining to an individual epidemic's parameters, while not extrapolating to an etiologic conclusion. Here, we utilize ranks rather than absolute values in order to aggregate historical mortality data – originally at varying scales of resolution from daily through monthly - from medieval “plagues” and modern bubonic and pneumonic plague outbreaks to a monthly resolution. We examine an aspect of the Black Death which has received growing attention in the literature during the last few years: the difference in the seasonality (month) of peak mortality between modern and historical plagues. We believe that using ranks of peak mortality addresses some of the issues regarding data uncertainty in historical epidemic documentation.

## Materials and Methods

### Quantifying Epidemiologic Data on Medieval and Modern Plagues

A number of ‘quasi-empirical’ datasets of Black Death mortality have now been transcribed from original documentation by various historians and epidemiologists, and are compiled here in this analysis. These include daily mortality at Givry, France [21] in 1348 during the primary epidemic wave (1347–1351) and St. Nizier-de-Lyon [22] at the beginning of the primary epidemic wave; daily mortality at Penrith, England, in 1597–8 [23] and Eyam, England, in 1665–66 [24], and weekly data from England and Wales after 1532, with the advent of ‘Bills of Mortality’ [25], presented here in its original weekly tabulated form (from 1639–47) and aggregated to a monthly resolution (for the years 1625 and 1666). Additional aggregated monthly mortality totals are also available for Marseille (France; 1720–21) [26], Debrecen (Hungary; 1739) [26], [27] and Moscow (1771) [26], [28].

**Figure 1:**
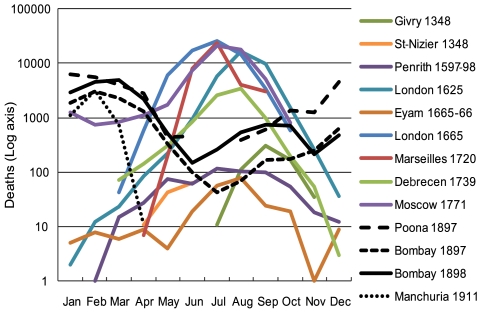
Monthly Mortality Data for the Medieval Black Death and later Plague Epidemics. Figure includes suspected Black Death epidemics through 1771, Bubonic Plague (Poona 1897, Bombay 1897, 1898), and Pneumonic Plague (Manchuria 1911). Monthly data include aggregated daily and weekly mortality records.

**Figure 2:**
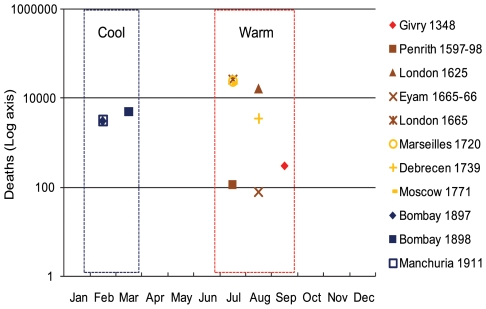
Monthly Peak Mortality for both Medieval Black Death and Bubonic and Pneumonic Plagues. Peak mortality among *Y. pestis*-variant diseases (bubonic and pneumonic plague) and suspected Black Death epidemics are inversely correlated by season.

**Figure 3:**
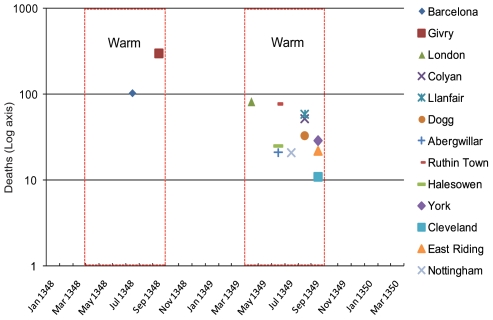
Monthly Peak Mortality for the Primary Wave of the Medieval Black Death: 1348–49. Barcelona data is based on ecclesiastical replacements, and London data is based on wills. The peak season of mortality is consistent for multiple locations during the time series.

Historical accounts, such as parish records, burial registers, Bills of Mortality, and documentary “plague tracts”, composed during the time of the Medieval Black Death (and extending through the Plague of Moscow in 1771), suggest that mortality peaked during the warm weather months between April and October. This is in stark contrast to pre-1347 monthly mortality records for England that show two peaks, one between January-February, and one between October-November [29]. This suggests a single peak replaced the pre-existing bimodal distribution of monthly deaths during the medieval Black Death [1]. Recent research results [1], [2], [30] also note this pattern of mortality and dissimilarities to observations of seasonal mortality maxima in laboratory-confirmed Y plague epidemics, which generally peak between November – April [2]. In this note, we compile monthly aggregated datasets of Y*. pestis* epidemics, in both bubonic [11] and pneumonic variants [12], and compare monthly peaks in mortality with those from 1348 [21], [22] and later suspected Black Death occurrences [17], [26]. (see Tables 1, 2: this includes the actual data on mortality for each outbreak included in Figure 1, 2 and 3).

**Table 1:** Recorded monthly mortality for MBD (and suspected MBD) epidemics.

Place	Date	Latitude	Deaths per month through the course of each epidemic wave												Agent
			Jan	Feb	Mar	Apr	May	Jun	Jul	Aug	Sep	Oct	Nov	Dec	
Givry^1^	1348	46°N	7						11	110	302	168	35		MBD
St. Nizier^2^	1348	45°N				11	43	63							MBD
Abergwillar^3^	1349	51°N			1			21	4	21	1				MBD
Colyan^3^	1349	51°N						10	25	52	12	4			MBD
Dogg^3^	1349	51°N			2			25	11	33	9	5			MBD
Llanfair^3^	1349	51°N						14	29	59	7	4			MBD
RuthinTown^3^	1349	53°N			2			77	14	61	4				MBD
Halesowen^4^	1349	52°N			2	3	21	25	22	3					MBD
Cleveland^5^	1349	53°N							1	7	11	7	2	1	MBD
East Riding^5^	1349	53°N							4	7	22	21	3	3	MBD
Nottingham^5^	1349	53°N					1		21	14	16	8	6	2	MBD
York^5^	1349	53°N				1	1	7	9	14	29	22	8	12	MBD
Penrith^6^	1598	54°N	0	1	15	27	75	61	115	103	101	55	18	13	*Plague*
London^7^	1625	52°N	2	12	23	85	224	954	5887	16455	9971	1514	256	36	*Plague*
Eyam^8^	1665	53°N	5	8	6	9	4	19	56	77	24	19	1	9	*Plague*
London^7^	1665	52°N			43	590	6137	17036	26230	14375	3449	590			*Plague*
Marseilles^9^	1720	43°N				7	200	8000	24000	4000	3000				*Plague*
Debrecen^9,10^	1739	47°N			72	145	313	824	2556	3493	998	195	54	3	*Plague*
Moscow^9^,^11^	1771	55°N	1240	744	851	1099	1708	7268	21401	17561	5235	805			*Plague*

**Agent and Highlights:**

MBD – Medieval Black Death; *Plague* – possible MBD.

**References:**

1 Gras (1939); 2 Biraben (1975); 3 Shrewsbury (1971); 4 Razi (1980); 5 Thompson (1911); 6 Scott (1995); 7 Creighton (1894, reprinted 1965); 8 Kendall (2000); 9 Alexander (1980, reprinted 2003); 10 Horvath (1962); 11 Shafonskii (1775, reprinted 1787).

**Table 2:** Recorded monthly mortality for Y. pestis plague epidemics. Note minima in warm season.

Place	Date	Latitude	Deaths per Month through the course of each epidemic wave												Agent
			Jan	Feb	Mar	Apr	May	Jun	Jul	Aug	Sep	Oct	Nov	Dec	
Bombay^12^	1897	19°N	1857	3083	2266	1316	328	99	42	70	168	178	255	644	BP
Bombay^12^	1898	19°N	2934	4498	4973	2171	524	150	263	543	731	728	217	470	BP
Manchuria^13^	1911	41°N	1104	3153	739	11									PP

**Agent and Highlights:**

BP – Bubonic Plague; PP – Pneumonic Plague.

**References:**

12 Indian Plague Commission Report; 13 Nishiura 2006.

We limit our analysis of Y*. pestis*-variant plague outbreaks to the widespread, highly lethal epidemics that hit Manchuria and India prior to 1920. Plague erupted in Manchuria in 1910–11 leaving ∼60,000 dead and again in 1920–21 killing ∼9300. Crowded inns and trains in winter provided optimal conditions for close personal contact and transmission of pneumonic plague, even though the basic reproductive number (R_0_) for both epidemics is ∼1 [15], [31]. Living conditions in Manchuria between 1910–1920 among the fur trappers who first contracted plague and the general population resemble the crowded, unsanitary conditions common throughout Europe during the medieval Black Death. Manchuria's humid continental climate is cold in winter during the fur trade's open hunting season [15], [31], [32]. In contrast, medieval Europe experienced pronounced seasonal trade and medieval fair activity that peaked in late summer/early fall, likely increasing transmission probabilities [1], [33], [34]. Previous comparative analysis of the propagation of the 1347–1351 primary wave of the medieval Black Death through Europe versus the propagation of plague through India established that: (1) medieval Black Death mortality was two orders of magnitude greater than plague in India; (2) modern Bubonic Plague is a rural disease while the medieval Black Death afflicted both urban and rural areas; (3) medieval Black Death propagation velocities were higher than those observed in India, even though India had modern railway transportation; and (4) the areal extent of the medieval Black Death was at a maximum in late fall while India's plague extent peaked in mid-late spring [2]. To reduce unnecessary confusion, we do not analyze the Sydney 1900–02 plague epidemic since Southern Hemisphere climatological seasons are inverted [13]. However, published reports on this Y*. pestis* epidemic are in agreement with our findings here; daily epidemic mortality peak occurred shortly after the coldest months of the year [13].

Since the 1920's, both bubonic and pneumonic plague epidemics have expanded beyond Asia to include cases in North and South America, Africa and Australia. Typically these epidemics are isolated. Repeated pneumonic plague outbreaks in Madagascar [14] tend to be ‘small, scattered, highly localized, and self-limiting’ [14], and bear no resemblance to the medieval Black Death, which had a broad, fast-moving epidemic front in the primary wave. Furthermore, recent work has noted that pneumonic plague is much less contagious that originally assumed, and that ‘bubonic plague never spreads directly from one person to another’ [15]. The most recent pneumonic plague outbreaks have occurred in tropical environments and therefore cannot be compared to seasonal, temperate outbreaks we analyze here.

Our objective was to validate the prior suggestions of inverse mortality peaks between medieval Black Death “plagues” and modern lab-confirmed Y*. pestis*-caused plague, and present a comprehensive monthly mortality meta-analysis of these epidemic data. Although calibrating historical data with modern observations and levels of laboratory confirmed accuracy is not possible, an analysis of the consistency of seasonal peaks in mortality among multiple data sets is possible. In this paper, we utilize data consistency in terms of seasonal peaks in mortality to evaluate data quality and identify consistent trends in peak mortality across spatial and temporal domains. In this manner, the likelihood of bias in one data set significantly altering our conclusions becomes less probable as additional data sets support or refute our original hypothesis.

## Results

### Identifying Seasonal Peaks in Mortality

Figure 1 illustrates the differences in monthly mortality between Y*. pestis* plague and suspected medieval Black Death epidemics. It is important to note that there are few complete records of lab-confirmed bubonic or pneumonic plague epidemics, and most of the records from such epidemics are from the late 1800's and early 1900's. Figure 1 includes weekly aggregated bubonic plague records from the Indian Plague Commission for Poona (1897) and Bombay (1897 and 1898). We also include daily aggregated data from the best-known pneumonic plague epidemic, which occurred in Manchuria in 1910 and 1911 and was originally collected by Japanese administrators in Manchuria and here supplied by H. Nishiura. Mortality records of the medieval Black Death in Givry, France in 1348 (probably the most complete daily dataset of mortality during the primary wave [30]) and St. Nizier-de-Lyon, France, in 1348 (a partial daily record of the primary epidemic wave [22]) is included, along with suspected Black Death outbreaks which occurred sporadically until 1771 (these are weekly data aggregated to month). Y*. pestis* plague is depicted in black and suspected Black Death outbreaks are depicted in color. The figure demonstrates that an inverse seasonal association exists between aggregated monthly Black Death mortality peaks and known Y*. pestis* plague. The data for these Y*. pestis* plague epidemics indicates a peak in lethality during the cool-months of the year (Nov. – Mar.) while the Black Death was more lethal during warm-months (Apr. – Oct.). It is also interesting to note that the Manchurian pneumonic plague epidemic (human-to-human versus the vector-borne transmission route of exposure which occurs in the bubonic variant) experiences a rapid decline in lethality as seasonal temperatures warm.

Figure 2 modifies Figure 1 to symbolize only the month of peak mortality. This figure clarifies the great differences in monthly peak mortality between Y*. pestis* plague (peak  =  February) and suspected Black Death outbreaks (peak  =  mid-July). Suspected Black Death epidemic mortality peaks during warm-weather months (red dashed box) and Y*. pestis* mortality peaks during cool-weather months (blue dashed box).

Figure 3 depicts mortality records of Black Death outbreaks during the primary wave of the medieval Black Death as it passed through Europe in 1348 and 1349. Included in Figure 3 are mortality peaks from Spain (Barcelona – based on ecclesiastical replacements [35]), France (Givry [21]), London (based on register of wills [36]), central England (deaths in Halesowen [37]) and the Dioese of York (actual clerical deaths from York, Cleveland, East Riding, Nottingham [38]), and Wales (deaths in Colyan, Llanfair, Dogg, Abergwillar, Rutin Town [39]). Both 1348 and 1349 exhibit peaks during two warm-weather seasons - occurring from April through October, and no location experiences a mortality peak during the cooler months of the year.

**Figure 4:**
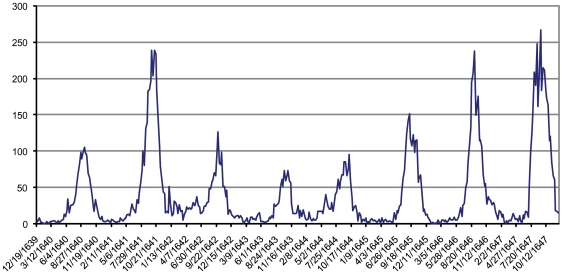
Annual Mortality Peaks Recorded in English “Bills of Mortality”: 1639–1647. Data depict an annual peak in mortality during the warmer months of the year. Only deaths specifically noted in the records as due to “plague” are included.

Figure 4 illustrates weekly plague mortality recorded in London from 1639–1647 [40]. This data does not depict any large-scale, widespread epidemic; rather the graph illustrates weak endemic mortality and the mortality peaks are consistent, occurring in the late summer through early fall.

In order to compare peak mortality between Y*. pestis* and suspected Black Death epidemics, we assign an ordinal value to the peak month of mortality in disparate epidemic data (Jan.  = 1−Dec.  = 12) and then utilize a two-sample difference of means test (2-tailed, unequal variances assumed). The mean peak month for Y*. pestis* mortality is month 2 (February) and is 7.4 (mid-July) for suspected Black Death – indicating a near-exact inverse correlation between the month of peak mortality between these diseases (5.4 months between peaks). As expected, we find that a statistical comparison between the peak mortality months in Y*. pestis* epidemics and suspected Black Death occurrences demonstrates a highly significant difference (p<0.001). Moreover, mortality peaks by month in Figure 2 are consistent over time and exhibit no significant difference between the peak epidemic months in suspected Black Death epidemics throughout the time series (p = 0.59). A statistically significant difference (p<0.001) also occurs between Y*. pestis* mortality peaks and aggregated 1348–49 peaks, even when additional data from districts of Bombay and Poona during the Indian bubonic plague outbreak of 1897–98 are included. Furthermore, a significant statistical difference (p<0.001) occurs between the medieval epidemic mortality peaks and Y*. pestis* mortality peaks which also includes data from widespread outbreaks in seasonal, northern hemisphere locations prior to 1953 (e.g., Burma, Vietnam, Thailand [41]). In summary, basic statistical t-tests between the peak months of Y*. pestis*-variant mortality and historical accounts of “plague” mortality indicate that they are undoubtedly different.

## Discussion

### Possible Explanations for Inverse Seasonal Peaks in Mortality

Previous works [2], [3], [5] have noted seasonal discrepancies in selected Black Death records of mortality versus observed Y*. pestis* outbreaks. Our analysis verifies these previous assertions with a multitude of compiled historical mortality data. We offer several possible explanations for the inverse seasonality of peak mortality. One possibility is that, if the Black Death was caused by Y*. pestis*-variant disease, then the characteristics of that organism, and perhaps its particular genetic strain, may differ from that which exists today [6]. Another possibility is that the route of exposure during the Black Death epidemics mimicked the route of exposure during modern plague outbreaks, albeit at different times of the year. A third option is the possibility of climate changes affecting disease transmission, but we discount this possibility due to the centuries across which suspected Black Death epidemics were recorded. Fourthly, there is the possibility that the Black Death was caused by a human-to-human virus [5], which would account for many of the factors noted in contrast to Y*. pestis*-variant plagues that are vector-borne. We suggest that a combination of factors may be to blame for the inverse seasonal mortality peaks [3]: perhaps not only was the etiologic agent of the Black Death something other than modern Y*. pestis*, but that market trade and religious pilgrimage during the medieval centuries allowed for human intermingling and disease transmission around the time when such activity occurred most – the warm-season. These possibilities require much more research, including interdisciplinary investigations, in order to validate any of these explanations.

### Significance

Reanalyzing the medieval Black Death and verifying its etiologic agent is becoming increasingly important, and not less so, in an age of rapid environmental and population changes. Similar changes were likely underway in the 1300's, and the primary wave of the Black Death came out of Asia and within a period of 4 years (1347–1351) killed anywhere from 30–60% of Europeans. Some event, perhaps a genetic mutation, climate shift, human-vector encounter, transportation network establishment, or a combination of environmental and human actions precipitated this deadly epidemic. As a result, we have begun analyzing historical “plague” records for as much information for reconsideration as possible. After all, our knowledge of pathogenic agents (including viruses, rickettsia, and prions, etc.) has increased exponentially since Yersin's [20] proclamation that the Black Death was an epidemic of bubonic plague.

The results presented here support prior research suggesting that there was an inverse seasonal peak in mortality between suspected Black Death epidemics and observations of Y*. pestis* plague mortality. The warm-season Black Death mortality peak is consistent for 420 years as illustrated in daily mortality data from Givry (1349) [21] to Moscow (1771) [26], [28]. This peak mortality is congruent with a seasonal peak in travel, trade and religious pilgrimage during this period [3]. We draw no specific etiologic conclusions from the results presented here, but do note that the frequency of published research questioning Y*. pestis* as the causative agent of the medieval Black Death has increased over the last decade. These findings add additional evidence toward future research into the deepening mystery of the medieval Black Death; however, this work represents only one of a number of investigational lines that may allow a reexamination of Yersin's original MBD paradigm going forward.
